# Comparative efficacy and safety of Cohen *versus* Lich-Gregoir ureteral reimplantation in pediatric vesicoureteral reflux: a systematic review and meta-analysis

**DOI:** 10.7717/peerj.20636

**Published:** 2026-02-06

**Authors:** Min Wang, Yu Xi, Nanxiang Huang, Li Zhang, Jinlong Liu

**Affiliations:** 1 Beijing Anzhen Nanchong Hospital of Capital Medical University & Nanchong Central Hospital, Nanchong, China; 2 Department of Operating Room, Nanchong Hospital of Traditional Chinese Medicine, Nanchong, China

**Keywords:** Vesicoureteral reflux, Cohen, Lich-Gregoir, Ureteral reimplantation, Meta-analysis

## Abstract

**Background:**

Cohen and Lich-Gregoir ureteral reimplantation techniques are the most commonly used surgical approaches for correcting vesicoureteral reflux (VUR) in children. While both techniques aim to restore the anti-reflux mechanism by lengthening the intramural ureter, their comparative efficacy and safety remain controversial. This meta-analysis aimed to systematically evaluate and compare the perioperative outcomes and postoperative complications of the Cohen and Lich-Gregoir procedures in pediatric patients with VUR.

**Methods:**

A systematic search of PubMed, Embase, Cochrane Library, CNKI, Wanfang, and VIP databases was conducted in May 2025 following PRISMA guidelines, registered under PROSPERO (CRD420251058493). Studies comparing Cohen and Lich-Gregoir techniques in pediatric VUR were included. Meta-analysis was performed using RevMan 5.3. Subgroup analyses were conducted for unilateral and bilateral VUR.

**Results:**

Eight retrospective studies involving 1,314 patients were included. Overall, the Lich-Gregoir technique was associated with shorter operative time (MD: 22.37 min, 95% CI [11.34–33.40]) and reduced hospital stay (MD: 2.65 days, 95% CI [1.59–3.71]). It also demonstrated lower risks of bladder spasms (OR: 5.93), hematuria (OR: 21.42), and overall complications. No significant differences were observed in postoperative catheter duration, persistent VUR, or urinary tract infection. Subgroup results were consistent with the overall findings.

**Conclusions:**

Compared to the Cohen technique, Lich-Gregoir reimplantation may offer advantages in operative efficiency and complication profile, especially in bilateral cases. These findings provide clinical insight but require further confirmation through high-quality prospective studies.

## Introduction

Primary vesicoureteral reflux (VUR) is one of the most common congenital urological anomalies in children, with an estimated incidence of 0.4%−1.8% (Chand et al., 2003; Tekgül et al., 2012). VUR is urine flowing retrograde from bladder to kidney. It is linked to recurrent urinary tract infections (UTIs) and renal scarring, and in severe cases to hypertension, renal dysfunction, and even end-stage renal disease (Gnech et al., 2024; Miyakita et al., 2020). The primary objective of treating VUR is to prevent renal injury and associated long-term complications.

Low-grade VUR can resolve or be managed with antibiotic prophylaxis, whereas surgery is warranted for high-grade reflux, breakthrough infections, or declining renal function. Over the past decades, various surgical techniques have been developed to correct VUR, including open, endoscopic, and minimally invasive approaches (Gnech et al., 2024; Miyakita et al., 2020). Among these, the Cohen cross-trigonal intravesical approach and the Lich-Gregoir extravesical approach are the two most widely performed methods in open ureteral reimplantation surgery (Austin & Cooper, 2004; Law et al., 2022). Both techniques aim to restore the anti-reflux mechanism by creating a submucosal tunnel to lengthen the intramural segment of the ureter, and both report excellent success rates ranging from 92% to 98% (Duckett, Walker & Weiss, 1992; Gnech et al., 2024).

Each method, however, presents distinct advantages and limitations. The Cohen technique is reliable and highly reproducible, suiting bilateral cases, but it may lead to hematuria, bladder spasm, longer catheterization, and difficult future upper-tract endoscopic access (Gillies et al., 2003; Valla et al., 2009). In contrast, the Lich-Gregoir approach offers less postoperative bladder irritation and a shorter recovery period, yet carries a risk of urinary retention due to pelvic plexus injury, particularly in bilateral surgeries (Castillo, Zubieta & Yanez, 2013; Palmer, 2008).

Although numerous single-center studies have explored the outcomes of Cohen and Lich-Gregoir procedures separately, direct comparisons between the two remain limited and inconclusive (Garcia Mérida et al., 2006; Li et al., 2022; Pan et al., 2024). Prior meta-analyses contrasted extravesical (EVUR) and intravesical (IVUR) as broad categories without distinguishing specific techniques (*e.g.*, Lich-Gregoir *vs* Cohen) or prespecifying laterality-based comparisons (Law et al., 2022). To address this gap, we synthesized evidence from eight retrospective studies directly comparing Cohen and Lich-Gregoir ureteral reimplantation in children and prespecified subgroup analyses by laterality (unilateral *vs* bilateral). To our knowledge, this meta-analysis is among the first to report laterality-based subgroup comparisons, providing decision-oriented, evidence-based guidance for surgical planning in pediatric VUR.

## Materials and Methods

This systematic review and meta-analysis was conducted in accordance with the Preferred Reporting Items for Systematic Reviews and Meta-Analyses (PRISMA) guidelines. The protocol was developed in advance and submitted to the PROSPERO database (University of York, UK) on 22 May 2025. The registration was confirmed on 23 May 2025 under the ID CRD420251058493. A preliminary feasibility screening of search results commenced on 22 May 2025 in parallel with protocol submission solely to refine scope and logistics; no data extraction or quantitative analysis was undertaken at that stage, and the prespecified inclusion/exclusion criteria were finalized before any eligibility decisions. Following registration confirmation, we re-screened all records against the prespecified criteria, completing title/abstract and full-text screening by 24 May 2025. Data extraction was performed from 25 to 31 May 2025.

### Literature search

A comprehensive literature search was conducted in May 2025 across six databases: PubMed, EMBASE, the Cochrane Library, CNKI, Wanfang, and VIP. Search terms included “vesicoureteral reflux”, “child”, “Cohen”, and “Lich-Gregoir”. There were no restrictions on publication language, and studies from January 2000 onward were considered. The detailed search strategies for each database are provided in Table S1. In addition, reference lists of relevant studies were manually searched to identify any additional eligible articles.

The included criteria were as follows: (1) included pediatric patients aged 0–18 years with primary VUR; (2) compared Cohen and Lich-Gregoir ureteral reimplantation techniques (unilateral or bilateral); (3) reported at least one of the predefined outcome measures.The criteria for exclusion were as follows: (1) studies not involving the relevant surgical interventions or lacking complete outcome data; (2) letters, case reports, reviews, and conference abstracts.

The selection of studies was independently performed by J.L.L. and Y.X. Discrepancies were addressed through mutual discussion, and unresolved conflicts were adjudicated by M.W., serving as the senior reviewer.

### Quality assessment

The methodological quality of the included studies was assessed using the Newcastle-Ottawa Scale (NOS), with a score ≥7 indicating high quality. Additionally, for non-randomized studies, the Risk of Bias in Non-Randomized Studies of Interventions (ROBINS-I) tool was used to evaluate bias across seven domains (Sterne et al., 2016). Two independent reviewers conducted the assessments, and discrepancies were resolved by consensus or third-party arbitration. To reduce bias, sensitivity analyses were conducted to explore the sources of heterogeneity, and studies with a high risk of bias were critically evaluated.

### Data extraction

Two reviewers independently extracted data from each eligible study using a standardized data collection form. Extracted information included study characteristics (first author, publication year, country, study design), patient demographics (sample size, age, laterality), surgical details (type of procedure, reflux grade), and clinical outcomes. The primary outcome measures were operative time (minutes), catheterization duration (days), and length of hospital stay (LOS) (days). Secondary outcomes included overall and specific postoperative complications such as UTI, bladder spasms, hematuria, and persistent vesicoureteral reflux. Discrepancies in data extraction were resolved through discussion or consultation with a third reviewer.

### Statistical analysis

Statistical analyses were performed using Review Manager (RevMan), Version 5.3. For continuous variables, mean differences (MDs) were calculated; for dichotomous variables, odds ratios (ORs)were used, both with 95% confidence intervals (CIs). Heterogeneity was assessed using the chi-squared test and the I^2^ statistic. If substantial heterogeneity was detected (I^2^ > 50%), a random-effects model was applied; otherwise, a fixed-effects model was used. A *p*-value < 0.05 was considered statistically significant. Subgroup analyses were conducted based on surgical laterality (unilateral *vs* bilateral cases). Comparisons were stratified to determine whether surgical outcomes differed between single-sided and bilateral VUR cases. The certainty of evidence for each main outcome was evaluated using the GRADE (Grading of Recommendations Assessment, Development and Evaluation) approach.

## Results

**Figure 1:**
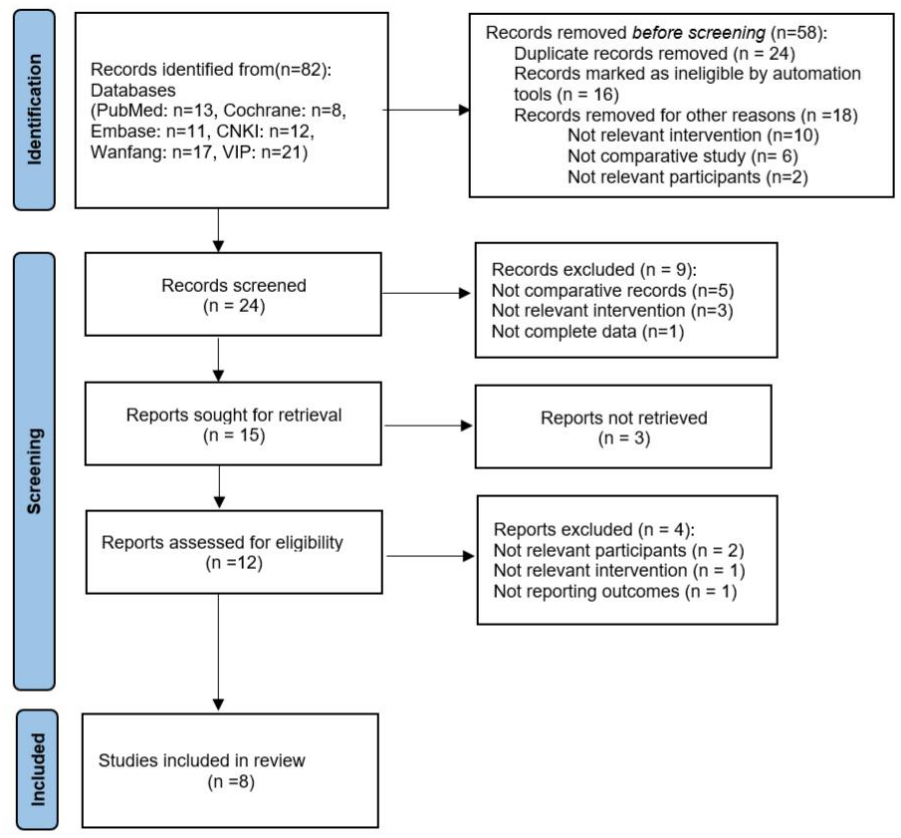
PRISMA flow diagram.

**Figure 2:**
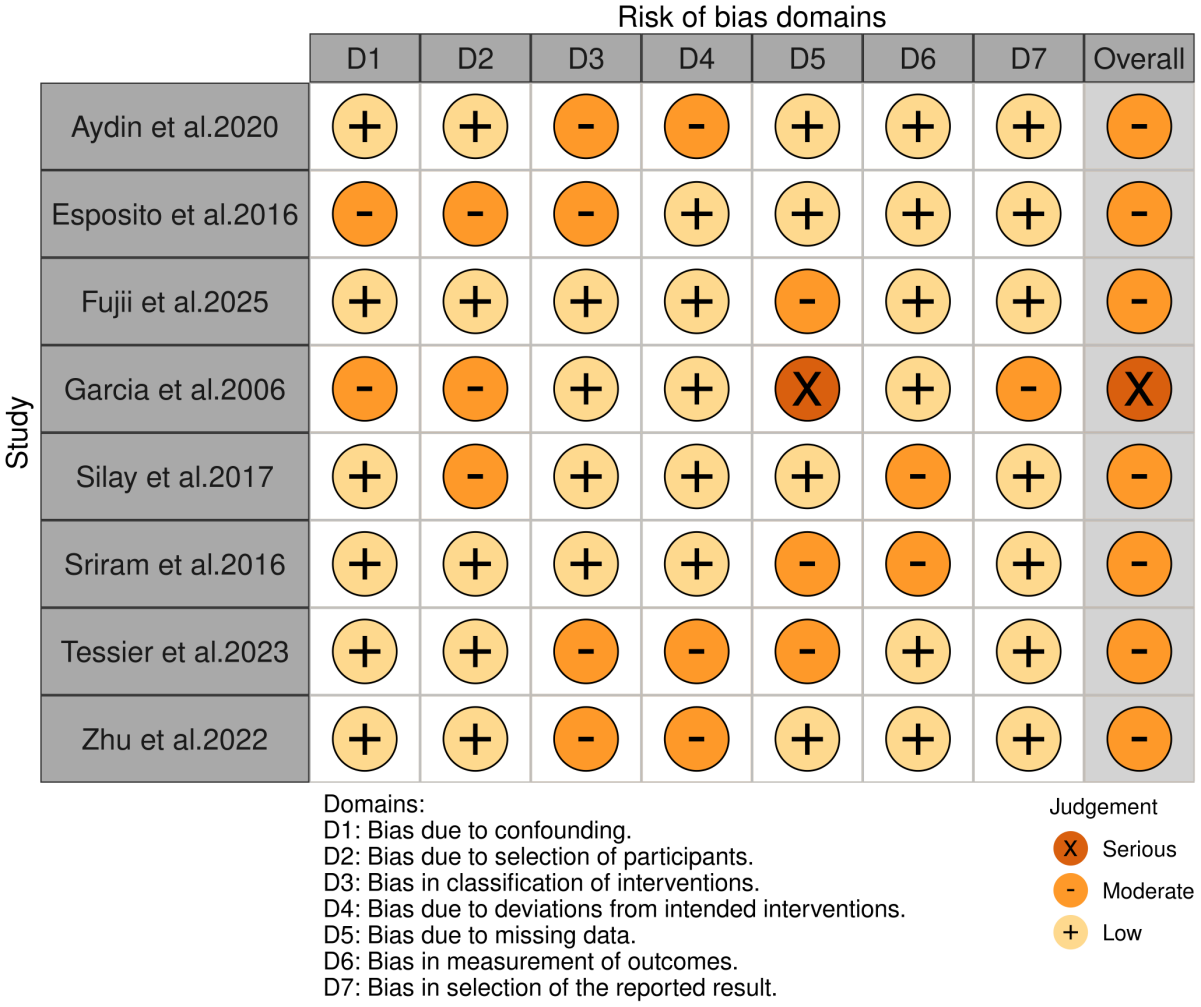
The risk of bias for the included studies according to ROBINS-I.

Following a rigorous screening process—including the removal of duplicates, exclusion of studies not aligned with the research question, and elimination of those with insufficient methodological quality or incomplete outcome reporting—a total of eight studies (Aydin et al., 2020; Esposito et al., 2016; Fujii et al., 2025; Garcia Mérida et al., 2006; Silay et al., 2018; Sriram & Babu, 2016; Tessier et al., 2023; Zhu et al., 2022) were deemed eligible for inclusion in the meta-analysis. The study selection process is illustrated in Fig. 1. Among the included studies, two focused exclusively on unilateral VUR (Aydin et al., 2020; Silay et al., 2018), two on bilateral VUR (Fujii et al., 2025; Sriram & Babu, 2016), one included (Zhu et al., 2022) both unilateral and bilateral subgroups, while the remaining three did not perform specific subgroup analyses based on laterality. Table 1 presents a comprehensive summary of the baseline characteristics and methodological quality of the included studies. Using ROBINS-I, we documented domain-level justifications for each judgment (confounding; selection; classification of interventions; deviations; missing data; outcome measurement; reporting) in Table S1. One study was rated high overall risk, mainly due to serious confounding (no adjustment for VUR grade/laterality or perioperative care) and inadequate outcome reporting; other studies were low to moderate risk across most domains. The traffic-light summary appears in Fig. 2.

**Table 1:** Characteristics and quality assessment of included studies.

Author, year	Study design	No.of patients	Age (years)	Laterality	Surgical approach	Reflux grade	Quality score^d^
Aydin et al. (2020)	R	18/20	3.4 ± 1.8/5.5 ± 2.9^a^	Unilateral/Unilateral	NA/NA	3.7 ± 0.8/3.9 ± 0.8	8
Esposito et al. (2016)	R	30/30	NA/NA	NA/NA	Open/Laparoscope	4.5 ± 0.6/3.5 ± 0.9	7
Fujii et al. (2025)	R	14/14	6.7(5.8–9.6)/6.5(5.4–7.8)^b^	Bilateral/ Bilateral	Open/Open	2.9 ± 0.9/2.6 ± 0.9	8
Garcia Mérida et al. (2006)	R	40/101	NA/NA	NA/NA	Open/Open	NA/2.9 ± 1	7
Silay et al. (2018)	R	23/35	4.6 ± 1.6/7.6 ± 4.2	Unilateral/Unilateral	Open/Open	3.4 ± 0.6/3.9 ± 0.5	8
Sriram & Babu (2016)	R	67/51	3(2–5)/1.25(0.83–2)^c^	Bilateral/Bilateral	Open/Open	NA/NA	8
Tessier et al. (2023)	R	77/35	3.8(1–11)/4(1–9.5) ^c^	NA/NA	Open/Laparoscope	NA/NA	7
Zhu et al. (2022)	R	22/25	7.8 ± 5.2/7.3 ± 4.7	Unilateral/Unilateral	Open/Robot	3.5 ± 0.5/3.48 ± 0.5	8
		18/22	4.7 ± 4.3/3.8 ± 2.5	Bilateral/ Bilateral	Open/Robot	3.47 ± 0.5/3.54 ± 0.5	

**Notes.**

R retrospective/Cohen versus Lich-Gregoir NA not available

a Mean ± Standard Deviation, SD.

b Median (Interquartile range, IQR).

c Mean (range).

d Using NOS scoring Rules.

### Overall comparison

**Figure 3:**
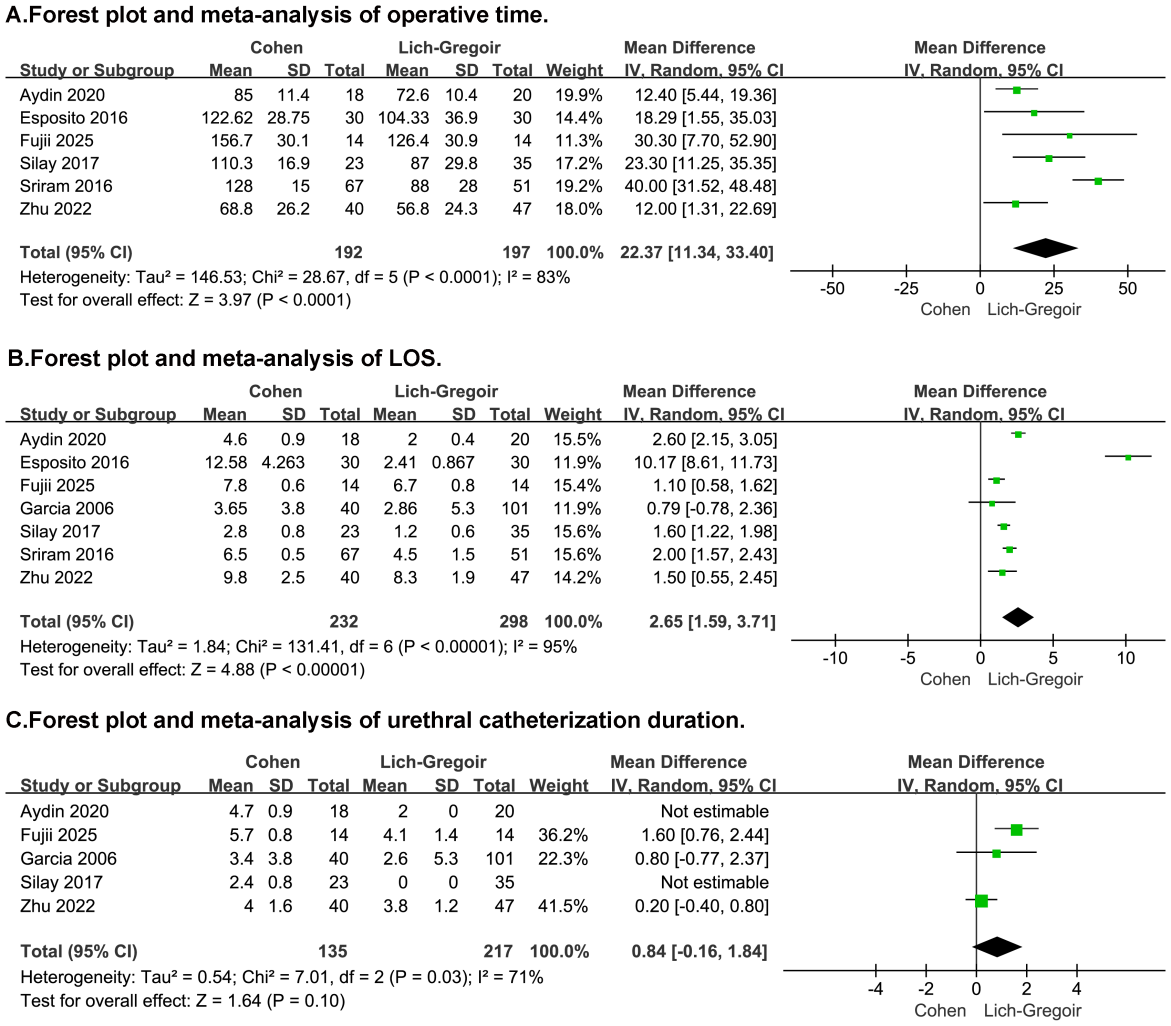
Forest plot and meta-analysis of operative outcomes: operative time (A), LOS (B), and urethral catheterization duration (C).

A total of eight retrospective studies were included to compare the efficacy and safety of Lich-Gregoir and Cohen ureteral reimplantation in pediatric VUR. In the overall analysis, the Lich-Gregoir technique demonstrated significantly shorter operative time (MD: 22.37, 95% CI [11.34–33.40], *p* < 0.0001, Fig. 3A) and LOS (MD: 2.65, 95% CI [1.59–3.71], *p* < 0.00001, Fig. 3B) compared to the Cohen approach. No statistically significant difference was observed in urethral catheterization duration (MD: 0.84, 95% CI [−0.16 to 1.84], *p* = 0.10, Fig. 3C).

**Figure 4:**
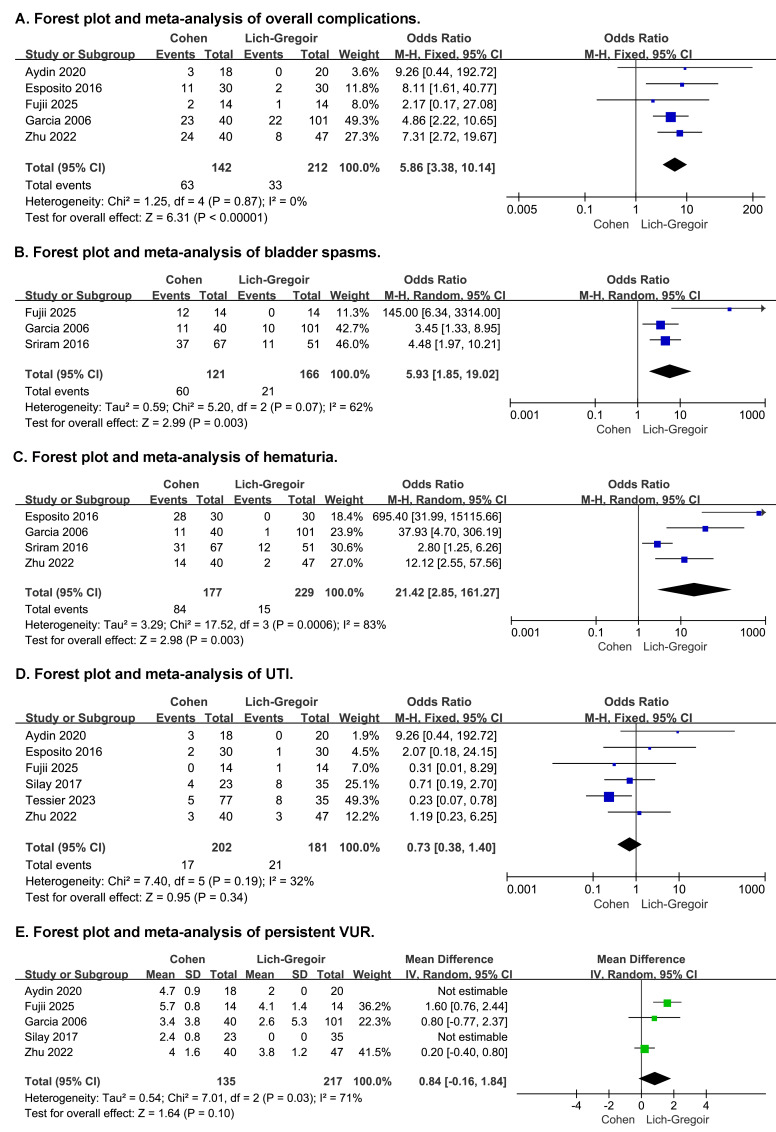
Forest plot and meta-analysis of postoperative complications: overall complications (A), bladder spasms (B), hematuria (C), UTI (D), and persistent VUR (E).

Regarding postoperative complications, Lich-Gregoir was associated with lower overall complication rates (OR: 5.86, 95% CI [3.38–10.14], *p* < 0.00001, Fig. 4A), including significantly reduced incidence of bladder spasms (OR: 5.93, 95% CI [1.85–19.02], *p* = 0.003, Fig. 4B) and hematuria (OR: 21.42, 95% CI [2.85–161.27], *p* = 0.003, Fig. 4C). The broad confidence interval for hematuria shows imprecision, likely due to small sample sizes and differences in reporting. However, no significant differences were observed in postoperative UTIs (OR: 0.73, 95% CI [0.38–1.40], *p* = 0.34, Fig. 4D) and persistent VUR (OR: 1.04, 95% CI [0.52–2.06], *p* = 0.92, Fig. 4E).

Substantial heterogeneity was detected in operative time, LOS, catheter duration, and bladder spasm outcomes (I^2^ >50%). Sensitivity analysis, conducted through sequential exclusion, identified the studies by Fujii et al. (2025) and Sriram & Babu (2016) as the main sources of heterogeneity. This was attributed to their inclusion of exclusively bilateral cases and surgeon-related biases respectively. The sensitivity analysis outcomes are detailed in Fig. S1. According to the GRADE assessment, the certainty of evidence was rated as very low for most outcomes, except hematuria, which was graded as low (Table S2).

### Unilateral VUR

In the subgroup of children with unilateral VUR, the Lich-Gregoir technique demonstrated a significantly shorter operation time compared to the Cohen procedure (MD: 12.78, 95% CI [9.51–116.06], *p* < 0.00001). Similarly, Lich-Gregoir was associated with a significantly reduced LOS (MD: 2.02, 95% CI [1.12–2.82], *p* < 0.00001). For overall postoperative complications, the Lich-Gregoir group showed a lower risk compared to the Cohen group (OR: 10.84, 95% CI [2.49–47.29], *p* = 0.002). However, no significant differences were observed in postoperative UTI (OR: 1.11, 95% CI [0.40–3.08], *p* = 0.83) or persistent VUR (OR: 1.70, 95% CI [0.07–38.98], *p* = 0.74) between the two approaches. The detailed results are presented in Table 2.

**Table 2:** Meta-analysis results for unilateral vesicoureteral reflux subgroup.

Outcomes	No. of studies	No. of patients	Heterogeneity			Merge analysis	
			Chi^2^	I^2^ (%)	*P* value	MD or OR (95% CI)	*P* value
Operation time	3	63/80	3.18	37	0.20	12.78 (9.51 to 116.06)	<0.00001
LOS	3	63/80	11.16	82	0.004	2.02 (1.12 to 2.82)	<0.00001
Overall complications	2	40/45	0.02	0	0.90	10.84 (2.49 to 47.29)	0.002
UTI	3	63/80	2.76	28	0.25	1.11 (0.40 to 3.08)	0.83
Persistent VUR	3	63/80	2.74	64	0.10	1.70 (0.07 to 38.98)	0.74

**Notes.**

LOS length of hospital stay UTI urinary tract infection VUR vesicoureteral reflux No. number/Cohen versus Lich-Gregoir Chi^2^ Chi-square test value I^2^ I-squared test value MD mean difference OR odds ratios 95% CI 95% confidence interval

### Bilateral VUR

In patients undergoing bilateral ureteral reimplantation, the Lich-Gregoir technique demonstrated a significant advantage in operation time compared to the Cohen approach (MD: 20.13, 95% CI [11.41–28.84], *p* < 0.00001). Similarly, the LOS was shorter in the Lich-Gregoir group (MD: 1.41, 95% CI [0.83–2.00], *p* < 0.00001). No statistically significant differences were observed in catheterization duration (MD: 0.82, 95% CI [−0.74 to 2.39], *p* = 0.30), urinary tract infection (OR: 1.17, 95% CI [0.25–5.51], *p* = 0.84), bladder spasm (OR: 14.99, 95% CI [0.29–786.95], *p* = 0.18), hematuria (OR: 3.20, 95% CI [0.82–12.44], *p* = 0.09), or persistent vesicoureteral reflux (OR: 0.92, 95% CI [0.27–3.11], *p* = 0.89). However, the overall complication rate remained significantly lower in the Lich-Gregoir group (OR: 5.20, 95% CI [1.54–17.55], *p* = 0.008). Results are presented in Table 3.

**Table 3:** Meta-analysis results for bilateral vesicoureteral reflux subgroup.

Outcomes	No. of studies	No. of patients	Heterogeneity			Merge analysis	
			Chi^2^	I^2^ (%)	*P* value	MD or OR (95% CI)	*P* value
Operation time	3	99/68	5.93	66	0.05	20.13 (11.41 to 28.84)	<0.00001
LOS	3	99/68	4.53	56	0.10	1.41 (0.83 to 2.00)	<0.00001
Catheterization duration	2	32/36	5.76	83	0.02	0.82 (−0.74 to 2.39)	0.30
Overall complications	2	32/36	0.63	0	0.43	5.20 (1.54 to 17.55)	0.008
UTI	2	32/36	0.93	0	0.34	1.17 (0.25 to 5.51)	0.84
Bladder spasm	2	81/46	6.05	83	0.01	14.99 (0.29 to 786.95)	0.18
Hematuria	2	85/54	2.12	53	0.15	3.20 (0.82 to 12.44)	0.09
Persistent VUR	2	85/54	0.13	0	0.72	0.92 (0.27 to 3.11)	0.89

**Notes.**

LOS length of hospital stay UTI urinary tract infection VUR vesicoureteral reflux No. number Cohen versus Lich-Gregoir Chi^2^ Chi-square test value I^2^ I-squared test value MD mean difference OR odds ratios 95% CI 95% confidence interval

## Discussion

Among available techniques, the Cohen intravesical and Lich-Gregoir extravesical ureteral reimplantations are most frequently performed, each with unique anatomical approaches and postoperative profiles (Gnech et al., 2024; Miyakita et al., 2020). While both achieve high reflux resolution rates, their comparative efficacy, complications, and recovery outcomes remain debated. This meta-analysis provides a timely synthesis to clarify their relative benefits and guide surgical decision-making.

Our meta-analysis indicates that both intravesical and extravesical ureteral reimplantation are highly effective in correcting pediatric VUR, with no significant difference in reflux resolution or postoperative hydronephrosis between techniques. This finding aligns with individual studies reporting equivalent success rates around 95–100% for Cohen and Lich-Gregoir repairs (Dogan et al., 2015; Esposito et al., 2016). However, clear differences emerged in surgical and recovery outcomes. The extravesical Lich-Gregoir approach was associated with significantly shorter operative times and briefer hospitalization. On pooled analysis, mean operative duration was about 23 min shorter for extravesical repairs. This corroborates multiple single-center series in which intravesical procedures took longer due to the technical steps of bladder incision and tunneling (Silay et al., 2018). Likewise, mean length of stay was roughly 2.65 days shorter with the extravesical approach in our meta-analysis, which may reflect faster postoperative recovery. Cohort studies consistently observed prolonged hospitalization after intravesical Cohen reimplantation owing to bladder-related discomfort and catheter needs (DeFoor & Hazelwood, 2014; Silay et al., 2018). Indeed, our included studies show that extravesical repair often permits minimal bladder catheterization. For example, Aydin et al. (2020) reported median urethral catheter durations of ∼2 days for Lich-Gregoir *vs.* ∼5 days for Cohen repairs, and others avoided any ureteral stents with extravesical surgery (Fujii et al., 2025). The ability to omit prolonged catheter drainage likely facilitates earlier mobilization and discharge in the extravesical group.

Postoperative complication profiles also differed in important ways. Children undergoing Cohen intravesical reimplantation experienced more bladder irritation symptoms, such as gross hematuria and bladder spasms, compared to those receiving the Lich-Gregoir repair. Intravesical surgery involves a bladder incision and often stent placement, which could help explain the higher incidence of hematuria and spasms (Ellsworth & Merguerian, 1995; Gillies et al., 2003). In our analysis, only the intravesical group routinely required ureteral stents or catheters, and consequently had a significantly higher rate of bladder spasms (one study noted 85.7% of Cohen patients had spasms *vs.* 0% with Lich-Gregoir, *p* < 0.001) (Fujii et al., 2025). These spasms can cause pain and require anticholinergic medications for relief (Ellsworth & Merguerian, 1995). Several studies also documented that postoperative analgesic requirements and pain scores were greater after the Cohen technique (Aydin et al., 2020; Esposito et al., 2016). For instance, Esposito et al. (2016) found that children in the open Cohen group had significantly worse pain and consumed more analgesics than those who underwent. Taken together, the evidence suggests the extravesical approach may afford a smoother immediate postoperative recovery, with less bleeding in the urine, less bladder spasm, and less need for pain control. Importantly, these benefits come without any compromise in surgical success. There were no differences in postoperative febrile UTI rates or reflux resolution, and both techniques had comparably low rates of ureteral obstruction or persistent reflux in the long term. Thus, for many scenarios, the Lich-Gregoir extravesical repair may offer offer faster convalescence and fewer early complications while achieving equivalent reflux correction to the Cohen intravesical technique. When persistent or downgraded reflux was observed after Lich-Gregoir, the reported management strategies included expectant observation with surveillance imaging (many cases remained low-grade and asymptomatic), endoscopic injection for symptomatic or persistent cases (*e.g.*, recurrent febrile UTI), and repeat reimplantation reserved for refractory or higher-grade failures (Dogan et al., 2015; Garcia Mérida et al., 2006; Gnech et al., 2024).

In our subgroup analyses, we further explored the comparative performance of the Cohen and Lich-Gregoir techniques in unilateral and bilateral VUR settings. In the unilateral subgroup, the Lich-Gregoir approach demonstrated advantages in operative time and length of hospital stay, consistent with overall findings. However, no significant differences were noted in postoperative catheter duration, overall complications, or persistent reflux, suggesting that both techniques offer comparable safety and effectiveness in unilateral cases. In contrast, bilateral VUR analysis revealed more distinct differences: the Lich-Gregoir group showed significantly reduced incidence of bladder spasms and hematuria, likely due to the avoidance of trigonal dissection inherent to extravesical access (Castillo, Zubieta & Yanez, 2013; Palmer, 2008). These findings highlight the potential patient comfort benefits of the Lich-Gregoir technique in bilateral cases, despite concerns about urinary retention. Notably, while bilateral application of the Lich-Gregoir procedure has raised concerns about transient urinary retention due to potential pelvic plexus injury, recent studies provide reassurance (Aydin et al., 2020; Burbige, Miller & Connor, 1996; Fujii et al., 2025; Fung et al., 1995). Where documented, retention was typically transient, resolving within hours to a few days with intermittent catheterization and conservative measures, without long-term voiding dysfunction. Fujii et al. (2025) reported that when the procedure was performed after toilet training, the incidence of urinary retention was markedly lower, likely due to the maturation of voluntary micturition reflexes and improved bladder control (Palmer, 2009). Moreover, nerve-sparing techniques based on detailed pelvic plexus anatomy may further reduce the risk of postoperative voiding dysfunction (David, Kelly & Poppas, 2004; Leissner et al., 2001). While both surgical approaches show similar success rates in bilateral VUR (around 90–100%), residual reflux after Lich-Gregoir was typically low-grade and did not necessitate reoperation or result in subsequent urinary tract infections. These findings support the consideration of patient age, toilet training status, and precise surgical technique as key modifiers when choosing the optimal approach for bilateral VUR correction.

Safety outcomes with sparse events-hematuria and bladder spasm-showed very wide confidence intervals, indicating substantial imprecision. This pattern likely reflects small sample sizes, low event rates, and heterogeneity in outcome definitions and reporting across studies. We therefore interpret these estimates cautiously as hypothesis-generating rather than practice-changing.

Beyond the pooled outcomes, insights from individual studies provide valuable context for clinical decision-making. For example, Fujii et al. (2025) noted significantly lower postoperative pain scores and reduced need for analgesics in the Lich-Gregoir group, suggesting enhanced postoperative comfort associated with the extravesical approach. Similarly, Esposito et al. (2016) and Silay et al. (2018) highlighted shorter time to ambulation and faster return to normal activity in patients undergoing Lich-Gregoir, underscoring functional recovery benefits. While cost analysis was not meta-analyzed due to limited reporting, Tessier et al. (2023) suggested that Lich-Gregoir may be associated with lower hospitalization costs due to shorter LOS and reduced catheter use. Conversely, Garcia Mérida et al. (2006) raised caution about the potential for transient postoperative urinary retention in bilateral Lich-Gregoir cases, emphasizing the importance of careful patient selection. Although not directly meta-analyzed, Zhu et al. (2022) reported parental satisfaction rates favoring Lich-Gregoir due to reduced visible hematuria and less irritative behavior postoperatively. Collectively, these findings complement our quantitative results and support the notion that the choice between surgical techniques should balance anatomic considerations with postoperative comfort, cost-efficiency, and patient-centered outcomes.

This meta-analysis has several limitations. First, all included studies were retrospective in design, introducing inherent risks of selection bias and unmeasured confounding. Second, heterogeneity was observed in key outcomes such as operation time and length of stay, likely reflecting differences in patient selection, surgeon experience, and perioperative protocols. Although sensitivity analyses were performed to explore potential sources of heterogeneity, residual confounding cannot be fully excluded. Third, the overall certainty of evidence, as assessed by the GRADE approach, was rated as low to very low for most outcomes, mainly due to study design limitations, inconsistency, and imprecision. Lastly, the limited number of studies, especially in subgroup comparisons, reduced the statistical power to detect small but clinically meaningful differences. These limitations should be considered when interpreting and applying the findings of this review.

## Conclusions

In conclusion, this systematic review and meta-analysis demonstrated that the Lich-Gregoir extravesical approach is associated with shorter operation time, reduced hospital stay, and fewer postoperative complications such as hematuria and bladder spasms compared to the Cohen intravesical technique in pediatric patients with vesicoureteral reflux. Lich-Gregoir may be preferred for improving perioperative outcomes and patient comfort, especially in bilateral VUR, helping with surgical planning. Subgroup analyses further suggest its consistent advantages in both unilateral and bilateral cases. Given the low to very low quality of evidence from retrospective designs and differences among studies, prospective trials are needed to confirm these findings and improve evidence quality.

##  Supplemental Information

10.7717/peerj.20636/supp-1 Supplemental Information 1 PRISMA checklist

10.7717/peerj.20636/supp-2 Supplemental Information 2 Flowchart

10.7717/peerj.20636/supp-3 Supplemental Information 3 Results of sensitivity analysis including operation time (A) LOS (B) urethral catheterization duration (C), and bladder spasm (D)

10.7717/peerj.20636/supp-4 Supplemental Information 4 Detailed search strategy used for database retrieval in PubMed, EMBASE, Cochrane Library, CNKI, Wanfang, and VIP

10.7717/peerj.20636/supp-5 Supplemental Information 5 GRADE evidence profile for outcomes included in the meta-analysis

10.7717/peerj.20636/supp-6 Supplemental Information 6 Rationale

10.7717/peerj.20636/supp-7 Supplemental Information 7 Raw data

## Additional Information and Declarations

### Data Availability

The following information was supplied regarding data availability: The raw data is available in the Supplemental File.
