# Supplementary material for: Comparative efficacy and safety of Cohen versus Lich-Gregoir ureteral reimplantation in pediatric vesicoureteral reflux: a systematic review and meta-analysis
Source: PeerJ. 2026 Feb 6;14:e20636. doi: 10.7717/peerj.20636 (PMC12884965; doi:10.7717/peerj.20636)
Supplement: Supplemental Information 2 [file peerj-14-20636-s002.docx]

**Identification of studies via databases and registers**

Records identified from(n=82):

Databases

(PubMed: n=13, Cochrane: n=8, Embase: n=11, CNKI: n=12, Wanfang: n=17, VIP: n=21)

Records removed *before screening* (n=58):

Duplicate records removed (n = 24)

Records marked as ineligible by automation tools (n = 16)

Records removed for other reasons (n =18)

Not relevant intervention (n=10)

Not comparative study (n= 6)

Not relevant participants (n=2)

Not comparative study(n=49)

Not original papers(n=38)

Not relevant patients(n=64)

**Identification**

Records excluded (n = 9):

Not comparative records (n=5)

Not relevant intervention (n=3)

Not complete data (n=1)

Records screened

(n = 24)

Reports sought for retrieval

(n = 15)

Reports not retrieved

(n = 3)

**Screening**

Reports excluded (n = 4):

Not relevant participants (n = 2)

Not relevant intervention (n = 1)

Not reporting outcomes (n = 1)

Reports assessed for eligibility

(n =12)

Studies included in review

(n =8)

**Included**

*Consider, if feasible to do so, reporting the number of records identified from each database or register searched (rather than the total number across all databases/registers).

**If automation tools were used, indicate how many records were excluded by a human and how many were excluded by automation tools.

*From:*  Page MJ, McKenzie JE, Bossuyt PM, Boutron I, Hoffmann TC, Mulrow CD, et al. The PRISMA 2020 statement: an updated guideline for reporting systematic reviews. BMJ 2021;372:n71. doi: 10.1136/bmj.n71

For more information, visit: <http://www.prisma-statement.org/>
