# Supplementary figures and images for: Comparative efficacy and safety of Cohen versus Lich-Gregoir ureteral reimplantation in pediatric vesicoureteral reflux: a systematic review and meta-analysis
Source: PeerJ. 2026 Feb 6;14:e20636. doi: 10.7717/peerj.20636 (PMC12884965; doi:10.7717/peerj.20636)

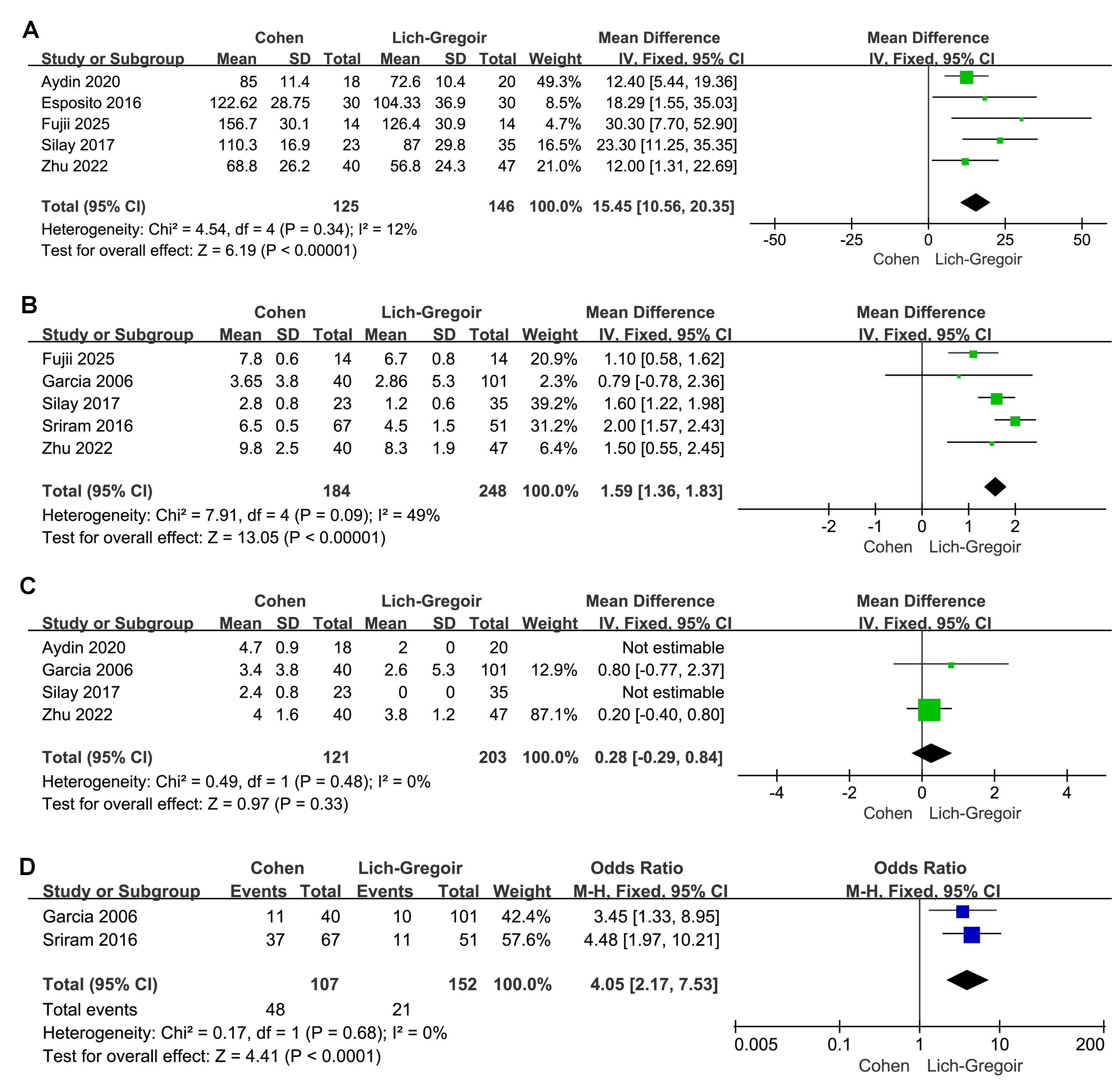

Supplement: Supplemental Information 3 [file peerj-14-20636-s003.png]
