# Supplementary material for: Comparative efficacy and safety of Cohen versus Lich-Gregoir ureteral reimplantation in pediatric vesicoureteral reflux: a systematic review and meta-analysis
Source: PeerJ. 2026 Feb 6;14:e20636. doi: 10.7717/peerj.20636 (PMC12884965; doi:10.7717/peerj.20636)
Supplement: Supplemental Information 4 [file peerj-14-20636-s004.docx]

**English databases**

**For Pubmed**

**ID**  **Search**

#1 vesicoureteral reflux [All Fields]

#2 vesico-ureteral reflux [All Fields]

#3 VUR [All Fields]

#4 Vesico-Ureteral Reflux[MeSH Terms]

#5 OR #1-4

#6 Cohen reimplantation [All Fields]

#7 cross-trigonal ureteral reimplantation [All Fields]

#8 Cohen cross-trigonal [All Fields]

#9 OR #6-8

#10 Lich-Gregoir [All Fields]

#11 Lich Gregoir technique [All Fields]

#12 OR #10-11

#13 child [MeSH Terms]

#14 pediatric [All Fields]

#15 children [All Fields]

#16 OR #13-15

#17 comparative study[Publication Type]

#18 comparative[Title/Abstract]

#19 meta-analysis[Publication Type]

#20 systematic review[Publication Type]

#21 OR #17-20

#22 #5 AND #9 AND #12 AND #16 AND #21

#23 Filters: from 2000/01/01

**For Embase:**

#1 'vesicoureteral reflux'/exp OR 'vesicoureteral reflux' OR 'VUR'

#2 'cohen reimplantation' OR 'cross trigonal reimplantation'

#3 'lich-gregoir'/exp OR 'lich gregoir' OR 'extravesical reimplantation'

#4 'ureteral reimplantation'/exp OR 'ureterovesical reimplantation'

#5 'child'/exp OR 'pediatric'/exp OR pediatric* OR child* OR children

#6 'comparative study'/exp OR 'meta-analysis'/exp OR 'systematic review'/exp OR comparative

#7 #1 AND #2 AND #3 AND #4 AND #5 AND #6

#8 Limit to: publication year ≥ 2000

**For Cochrane Library:**

#1 vesicoureteral reflux OR VUR OR vesico-ureteral reflux

#2 Cohen reimplantation OR cross-trigonal ureteral reimplantation

#3 Lich-Gregoir OR Lich Gregoir technique OR extravesical ureteral reimplantation

#4 ureteral reimplantation OR ureterovesical reimplantation

#5 child OR pediatric OR children

#6 meta-analysis OR systematic review OR comparative study

#7 #1 AND #2 AND #3 AND #4 AND #5 AND #6

#8 Publication Year from 2000 to 2025

**Chinese databases**

**For CNKI**

主题 = ("膀胱输尿管返流" OR "膀胱输尿管反流") AND

(主题 = "Cohen术式" OR "Cohen输尿管再植术" OR "跨三角输尿管再植术") AND

(主题 = "Lich-Gregoir术式" OR "Lich-Gregoir输尿管再植术" OR "膀胱外输尿管再植术") AND

(主题 = "儿童" OR "小儿")

文献类型 = "期刊"

时间范围：2000年–现在

**For Wanfang**

检索式：

(膀胱输尿管反流 OR 膀胱输尿管返流) AND

(Cohen术式 OR 跨三角输尿管再植术) AND

(Lich-Gregoir术式 OR 膀胱外输尿管再植术) AND

(儿童 OR 小儿)

限制条件：

- 文献类型：期刊

- 发表时间：2000年及以后

**For VIP**

主题 = (“膀胱输尿管反流” OR “膀胱输尿管返流”) AND

(“Cohen术式” OR “跨三角输尿管再植术”) AND

(“Lich-Gregoir术式” OR “膀胱外输尿管再植术”) AND

(“儿童” OR “小儿”)

限定：期刊；发表时间 ≥ 2000年
