# Supplementary material for: Comparative efficacy and safety of Cohen versus Lich-Gregoir ureteral reimplantation in pediatric vesicoureteral reflux: a systematic review and meta-analysis
Source: PeerJ. 2026 Feb 6;14:e20636. doi: 10.7717/peerj.20636 (PMC12884965; doi:10.7717/peerj.20636)
Supplement: Supplemental Information 5 [file peerj-14-20636-s005.pdf]

Author(s):

Question: Cohen compared to Lich-Gregoir for VUR

Setting:

Bibliography:

Certainty assessment							№ of patients		Effect		Certainty	Importance
№ of studies	Study design	Risk of bias	Inconsistency	Indirectness	Imprecision	Other considerations	Cohen	Lich-Gregoir	Relative (95% CI)	Absolute (95% CI)		
**operative time**												
6	non-randomised studies	extremely serious[a]	not serious	not serious	not serious	publication bias strongly suspected[b]	192	197	-	MD **22.37 higher** (11.34 higher to 33.4 higher)	⨁◯◯◯ Very low[a,b]	CRITICAL
**length of hospital stay**												
7	non-randomised studies	very serious[a,c]	not serious	not serious	not serious	publication bias strongly suspected[b]	232	298	-	MD **2.65 higher** (1.59 higher to 3.71 higher)	⨁◯◯◯ Very low[a,b,c]	CRITICAL
**catheterization duration**												
5	non-randomised studies	very serious[a,d]	not serious	not serious	serious[c]	publication bias strongly suspected[b]	135	217	-	MD **0.84 higher** (0.16 lower to 1.84 higher)	⨁◯◯◯ Very low[a,b,c,d]	CRITICAL
**postoperative complications**												
5	non-randomised studies	very serious[a]	not serious	not serious	serious[c]	publication bias strongly suspected[b]	63/142 (44.4%)	33/212 (15.6%)	**OR 5.86** (3.38 to 10.14)	**364 more per 1,000** (from 228 more to 496 more)	⨁◯◯◯ Very low[a,b,c]	CRITICAL
**urinary tract infection**												
6	non-randomised studies	very serious[a,c]	not serious	not serious	not serious	publication bias strongly suspected[b]	17/202 (8.4%)	21/181 (11.6%)	**OR 0.73** (0.38 to 1.40)	**29 fewer per 1,000** (from 69 fewer to 39 more)	⨁◯◯◯ Very low[a,b,c]	CRITICAL
**bladder spasms**												
3	non-randomised studies	extremely serious[a,e]	not serious	not serious	serious[e]	publication bias strongly suspected[b]	60/121 (49.6%)	21/166 (12.7%)	**OR 5.93** (1.85 to 19.02)	**336 more per 1,000** (from 85 more to 607 more)	⨁◯◯◯ Very low[a,b,e]	IMPORTANT

Certainty assessment							№ of patients		Effect		Certainty	Importance
№ of studies	Study design	Risk of bias	Inconsistency	Indirectness	Imprecision	Other considerations	Cohen	Lich-Gregoir	Relative (95% CI)	Absolute (95% CI)		
**hematuria**												
4	non-randomised studies	serious[f]	not serious	not serious	not serious	publication bias strongly suspected[b]	84/177 (47.5%)	15/229 (6.6%)	**OR 21.42** (2.85 to 161.27)	**535 more per 1,000** (from 101 more to 853 more)	⨁⨁◯◯ Low[b,f]	IMPORTANT
**persistent vesicoureteral reflux**												
6	non-randomised studies	very serious[a,d]	not serious	not serious	not serious	publication bias strongly suspected[b]	17/218 (7.8%)	20/284 (7.0%)	**OR 1.04** (0.52 to 2.06)	**3 more per 1,000** (from 33 fewer to 65 more)	⨁◯◯◯ Very low[a,b,d]	CRITICAL

CI: confidence interval; **MD:** mean difference; **OR:** odds ratio

Explanations

a. Differences in surgical laterality and approach.

b. Computer-based literature searching could not include all relevant studies. And grey literature also could not be included.

c. Postoperative management bias of different institutions.

d. Surgeon experience

e. Small sample

f. Differences in surgical approach.
