# Supplementary material for: Comparative efficacy and safety of Cohen versus Lich-Gregoir ureteral reimplantation in pediatric vesicoureteral reflux: a systematic review and meta-analysis
Source: PeerJ. 2026 Feb 6;14:e20636. doi: 10.7717/peerj.20636 (PMC12884965; doi:10.7717/peerj.20636)
Supplement: Supplemental Information 6 [file peerj-14-20636-s006.docx]

**Rationale:**

Ureteral reimplantation is a common surgical procedure for pediatric patients with vesicoureteral reflux (VUR) and other lower urinary tract abnormalities. Among the commonly used extravesical approaches, the Cohen and Lich-Gregoir techniques are both effective but differ in surgical route, complexity, and postoperative profiles. Although several retrospective studies have compared the two, inconsistent findings regarding operative time, complication rates, and recovery duration have led to clinical uncertainty. Additionally, no previous meta-analysis has systematically evaluated the comparative outcomes of these two techniques across unilateral and bilateral cases. A rigorous synthesis of existing evidence is urgently needed to better inform surgical choices in pediatric urology.

**Contribution to Knowledge:**

This study provides the first comprehensive meta-analysis comparing the Cohen and Lich-Gregoir ureteral reimplantation techniques in children, with subgroup analyses for unilateral and bilateral VUR cases. It quantifies differences in operative metrics and complication profiles and highlights surgical considerations not captured in prior individual studies. By consolidating fragmented evidence and assessing the quality of findings via GRADE, the study offers valuable insights to guide clinical decision-making and may inform future surgical guidelines in pediatric urology.
